# Late Development of Large Fœtus in Abdomen of an Adolescent of the Masculine Sex

**Published:** 1896-06

**Authors:** 


### LATE DEVELOPMENT OF LARGE FŒTUS IN ABDOMEN OF AN ADOLESCENT OF THE MASCULINE SEX.

(Academy of Medicine, Paris, France, meeting of 5th May, 1896.)

Dr. M. G. Lévy read observations made by Drs. Maydi and Sänger, of Prague, on a young man of nineteen years, who had suffered for two years from an abdominal tumor, which slowly acquired the size of the head of an infant at term. This tumor was situated under the peritoneum and between the folds of the mesentery. The patient died twenty-four hours after the operation for its removal.

The tumor contained a yellowish, gelatinous liquid and a well-developed fœtus, feminine sex, apparently of fifth month of gestation. Dr. Lévy states that "cysts" of this nature exist generally from birth, and receive a growth impulse, more or less strong, at the period of puberty of patient, or from a traumatism.—*Semaine Medicale, Paris.*

